# Supplementary material for: Vitamin D Modified DSS-Induced Colitis in Mice via STING Signaling Pathway
Source: Biology (Basel). 2025 Jun 18;14(6):715. doi: 10.3390/biology14060715 (PMC12190092; doi:10.3390/biology14060715)
Supplement: Supplementary file 1 [file biology-14-00715-s001.zip › Supplementary Materials S1: 16s-report of gut microbiota/Groups/otuVenn/upset.pdf]

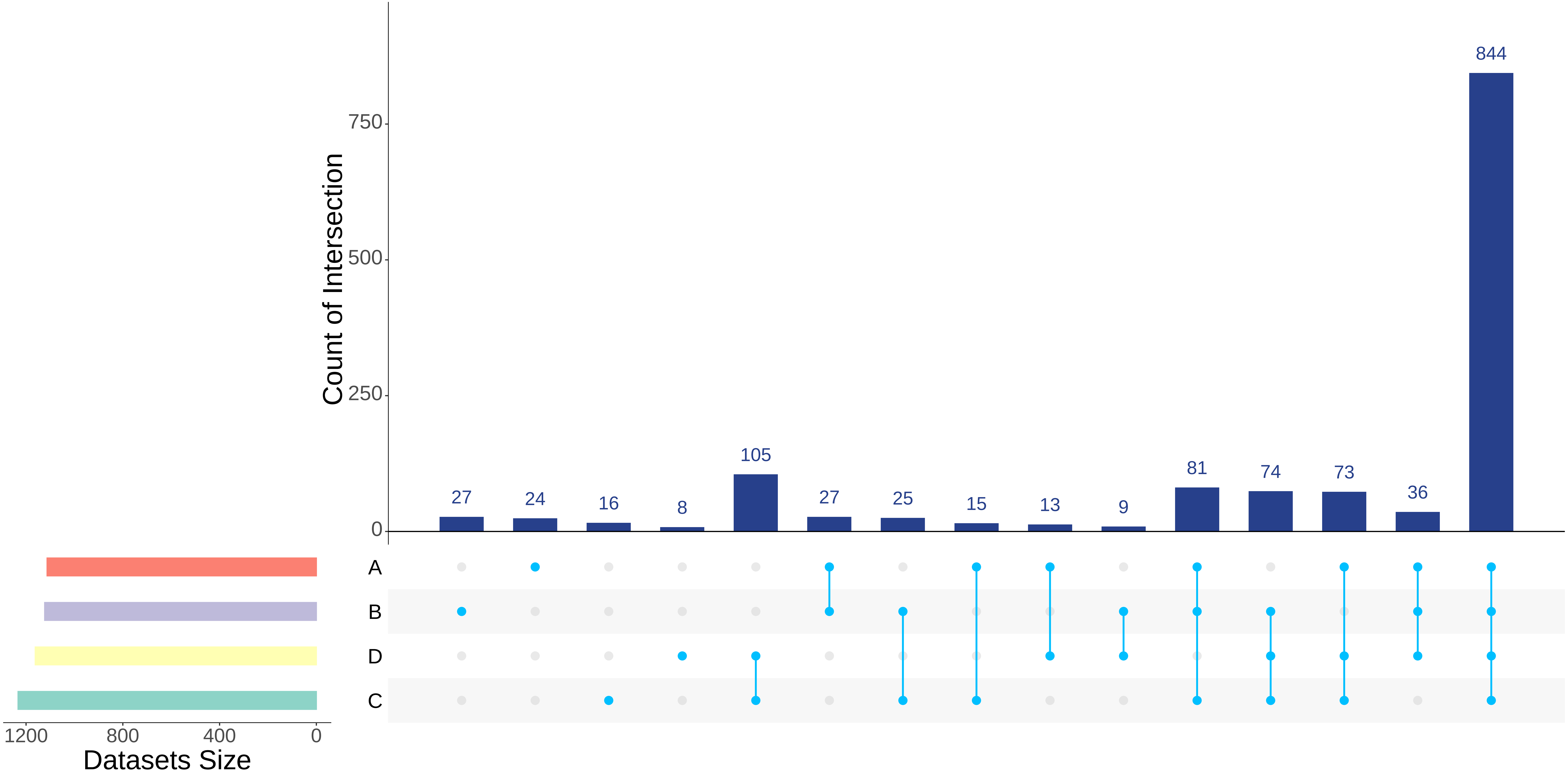

Count of Intersection
0
250
500
750
27
24
16
8
105
27
25
15
13
9
81
74
73
36
844
A
B
D
C
1200
800
400
0
Datasets Size
